# Does digital access translate into human capital gains? Assessing information technology use effects on cognitive and non-cognitive development of students in Western Rural China

**DOI:** 10.1371/journal.pone.0349438

**Published:** 2026-06-01

**Authors:** Bin Tang, Zeming Cheng, Xiaoli Lin, Yunxuan Cao, Siyi Xiao, Yunhui Ma, Kaixuan Gao

**Affiliations:** 1 Center for Experimental Economics in Education, Faculty of Education, Shaanxi Normal University, Xi’an, Shaanxi, China; 2 Beijing Friendship Hospital, Capital Medical University, Beijing, China; 3 The Library, Shaanxi Normal University, Xi’an, Shaanxi, China; BRAC University, BANGLADESH

## Abstract

This study explores how the use of information technology (IT) influences the development of cognitive and non-cognitive abilities among primary school students in rural areas of western China. As IT tools become more integrated into daily life, especially in underdeveloped regions, their influence on education has grown significantly. Relying on Heckman’s human capital development theory, we apply Propensity Score Matching combined with a Difference-in-Differences (PSM-DID) approach to analyze two rounds of panel data collected from third and fourth grade students between 2019 and 2020. The analysis suggests that while IT use does not significantly boost cognitive skills, a slight positive trend is observed, likely reflecting the coexistence of both beneficial and adverse effects. In terms of non-cognitive skills, frequent IT use appears to enhance students’ openness and increase their fondness for school and teachers, yet it is also linked to rising levels of self-blame and anxiety. Additional findings show that the effects of IT use are more pronounced among older children, Han Chinese students, and those from households with lower income or lower parental education. These results contribute to a deeper understanding of IT’s role in shaping human capital in rural settings and offer practical implications for policymakers. In particular, they highlight the need to maximize the positive aspects of IT in promoting equity in education while addressing its potential downsides through more customized support for diverse student populations.

## 1. Introduction

The fast-paced development of information technology is changing how education works, especially in less developed regions [1]. In recent years, researchers have paid more attention to how IT affects students’ cognitive and non-cognitive abilities [2,3]. Technologies like smartphones, virtual reality tools, and online learning platforms are increasingly used to support students’ cognitive progress. For example, Khan and colleagues reported that these digital tools could improve students’ attention, memory, problem-solving ability, and critical thinking by making the learning process more interactive and engaging [4]. Their findings echo the views of Gheith and Aljaberi [5], who pointed out the importance of building non-cognitive traits such as perseverance and self-confidence, which are closely linked to students’ academic growth and preparation for the job market. Similarly, Beuermann et al. [6] found in Peru that giving students laptops helped them improve digital literacy. In another case, Fajebe et al. [7] observed that mobile learning in Rmwada played a role in improving students’ reading skills in English. Taken together, these studies suggest that IT use has real potential to support education in developing countries and also raise the need for more detailed studies on how it shapes students’ overall development.

According to Heckman’s theory of human capital development, both cognitive and non-cognitive abilities are crucial for an individual’s success in the labor market and society [8–10]. The education production function suggests that these abilities, which are education outputs, can be influenced by factors such as students’ characteristics, family background, school environment, teacher quality, and other educational inputs [11,12]. Although information technology is not explicitly mentioned in education production function, its impact on all aspects of education cannot be ignored in today’s highly technological world [13,14], especially in developing countries where it may have a more significant effect on the education production process [15]. From the perspective of Heckman’s human capital framework, cognitive and non-cognitive skills are jointly produced during childhood through the interaction of individual traits, family environment, school inputs, and other developmental investments. In this sense, information technology can be understood as a contemporary educational input that may influence children’s human capital formation through multiple channels. This perspective provides the theoretical basis for examining IT use not only in relation to academic performance, but also in relation to broader non-cognitive outcomes.

The growing use of information technology in children’s lives brings both benefits and risks. By the end of 2023, the proportion of internet users among urban and rural minors in China reached 97.5% and 96.5%, and about 95.1% of primary school students had access to the internet [16]. This shows that digital tools have become a regular part of many students’ daily routines. The outbreak of COVID-19 further pushed the role of technology in education, making it an important tool not only for studying but also for entertainment and staying connected with others [17]. On the positive side, better internet access, improved data tools, and AI-based platforms have allowed students to reach more learning materials [14], try personalized learning methods [18], and explore creativity and innovation in new ways [19,20]. At the same time, there are worries about how too much time online might affect students’ privacy and safety [20], their physical and mental health [21], and their ability to socialize and develop proper values [22].

Recent evidence also suggests that children’s digital engagement should not be treated as a single undifferentiated behavior. A 2025 OECD report shows that children and adolescents use digital devices for multiple purposes, including entertainment, social networking, communication, information seeking, and content creation, with substantial variation across contexts and countries. Likewise, UNICEF’s 2025 report emphasizes that digital technologies may affect children positively or negatively depending on how they are used and what kinds of content and experiences they involve. These newer findings reinforce the need to move beyond aggregate measures of IT exposure and instead distinguish among different use types when assessing developmental outcomes.

Given the growing interest in how information technology can support human capital development in rural settings, this paper looks into the effects of IT use on both cognitive and non-cognitive development among primary school students in western China. The study contributes to the existing research in four key ways. First, it focuses on an often-overlooked region, rural western China, where limited educational resources and ethnic diversity create a very different setting from the urban or more developed areas usually studied. By providing empirical evidence from these underserved communities, this work helps fill an important gap in the literature on educational technology in developing countries.

Second, it empirically and theoretically extends existing theories by combining Heckman’s theory of human capital education with technology-enhanced education. Compared to the mainstream literature focusing on cognitive performance, our multidimensional measure of technology-enabled learning empowers us to examine how IT affects students’ performance in terms of cognitive skills and non-cognitive skills, thus expanding the debates on how IT will be beneficial for children’s well-rounded growth.

Third, technically, the study strengthens rigorous causal inference by employing a PSM-DID design that controls for endogenous biases in the observational studies on rural education. Taking the power of combining propensity score matching with a longitudinal difference-in-differences analysis on a two-wave panel (2019–2020) data, we estimate the net effects of IT exposure for eliminating confounding effects from self-selection biases.

Lastly, the findings offer new insights that challenge some common assumptions. Although IT appears to have no statistically significant impact on cognitive outcomes, the direction is mostly positive—possibly because better access to information is balanced out by the distractions digital tools can also bring. In terms of non-cognitive development, IT has both benefits and drawbacks: it helps students become more open and feel more connected to school, but it also leads to increased anxiety and self-blame in some cases. These mixed results highlight the importance of looking closely at different student groups. In fact, the effects of IT are stronger among older children, Han Chinese students, and those from poorer or less educated families. Together, these findings suggest that while IT holds promise for reducing inequality in education, its implementation needs to be tailored to the needs of different groups in resource-limited areas.

## 2. Literature review

### 2.1. Concept definition

#### 2.1.1. Concept of cognitive ability.

Cognitive ability refers to a person’s capacity to learn, remember, and use information. According to Kautz et al. [23], it includes different aspects such as intelligence, memory, problem-solving, and language skills. In recent years, large-scale international tests like the Programme for International Student Assessment (PISA) and the Trends in International Mathematics and Science Study (TIMSS) have been widely used to assess student abilities in areas like reading, language, math, and science within educational systems.

Strong cognitive skills not only help students perform better in school [24], but are also closely linked to future job opportunities and earnings [25]. Cognitive abilities are influenced by many different factors, including a child’s personal traits, family background, school environment, and the broader social and cultural context they grow up in [26,27]. Understanding how these elements work together to shape cognitive development is important for helping students succeed in both education and future careers.

#### 2.1.2. Concept of non-cognitive ability.

Non-cognitive ability, also known as social and emotional ability, refers to a person’s capacity to manage emotions, set and work toward goals, understand others’ perspectives, build positive relationships, make thoughtful choices, and handle social situations effectively. Along with cognitive ability, non-cognitive ability forms an essential part of a person’s human capital. The OECD framework highlights how these non-academic traits can influence overall competence and development [28,29].

In 1997, the Collaborative for Academic, Social and Emotional Learning (CASEL) outlined key areas of non-cognitive ability, including self-awareness, self-management, social awareness, communication skills, and responsible decision-making [29]. Since then, these skills have received growing attention in educational and psychological research. In 2017, the OECD launched the Survey on Social and Emotional Skills (SSES), which aimed to build a framework to assess such abilities using tools like the Big Five Personality Test. The Internal-External Control Scale and the Self-Esteem Scale, which are known general measures, were also taken into consideration for measuring the skills [29].

Studies show that non-cognitive skills are linked to various life outcomes such as academic performance [29], employment prospects [9], overall well-being [30], and health [31]. Some of these skills may decline with age [29]. In addition, family-related factors like parents’ education and household income [32], as well as the sense of belonging at school and teacher-student relationship quality [29], are strongly associated with the development of non-cognitive abilities.

In this paper, mental health is considered part of non-cognitive development because children’s emotional well-being, anxiety control, impulse regulation, and self-related emotional responses are closely connected to broader social-emotional skills. While mental health is not identical to non-cognitive ability, it reflects an important aspect of students’ emotional adjustment and behavioral regulation, both of which are central to the development of non-cognitive capacities in childhood.

#### 2.1.3. Concept of information technology.

Information technology refers to a broad range of technical tools and resources that are used to transmit, produce, publish, store, and manage information [33]. In recent years, Chinese students under the age of 18 have increasingly used information technology for learning, entertainment, and social communication [34,35].

In education, the progress of informatization has introduced new forms of interaction and enriched learning materials for students. These changes have helped improve learning outcomes and allowed students to become more independent in their studies [36]. At the same time, some scholars have pointed out that data-based learning models may bring unintended problems for academic development [37,38]. For entertainment activities, the use of graphic software, multimedia devices, and gaming platforms has expanded students’ horizons and encouraged creative thinking [39]. On the downside, internet addiction and a lack of ethical boundaries in virtual environments have been seen as harmful to adolescent growth [40]. In the area of social interaction, information technology allows young people to create and share content more easily. This practice helps them build better communication and teamwork skills, and strengthens their awareness of social relationships [41,42]. However, researchers have also raised concerns about data privacy, digital security, and ethical behavior online, which are becoming more urgent and need serious attention from society [43].

Overall, information technology has become an important part of young people’s learning, entertainment, and social lives. While it brings many benefits, it is also important to carefully address the risks and challenges it creates, so that adolescents can grow up in a balanced and healthy way.

In this study, we distinguish IT use into three functional domains—learning, entertainment, and socializing—because these represent the most common purposes for which primary school students engage with digital devices in daily life. More importantly, these domains expose children to different types of content, require different forms of engagement, and may affect developmental outcomes through different mechanisms. Learning-oriented use is more directly related to academic tasks and knowledge acquisition; entertainment-oriented use is more closely linked to leisure consumption and distraction; and social-oriented use primarily concerns communication and peer interaction. Distinguishing among these domains therefore helps avoid treating IT use as a homogeneous construct and allows us to examine whether different patterns of digital engagement are associated with different developmental consequences.

### 2.2. Literature review

#### 2.2.1. Literature on the impact of information technology use on cognitive ability.

Some researchers believe that using information technology (IT) can help improve students’ cognitive development. Many studies have shown that IT use in both education and entertainment has a positive effect on abilities like reading and math [44–48]. In addition, studies focusing on how often and how long students use IT suggest that more frequent use is linked to better cognitive skills [48,49]. Some experimental research, including randomized controlled trials, has also found that programs involving the use of hardware or software can improve cognitive ability, with effects around 0.4 standard deviations [50]. More recent studies continue to support these results, showing that digital learning tools play a role in boosting students’ thinking and learning skills [51,52].

#### 2.2.2. Literature on the impact of information technology use on non-cognitive ability.

Studies on non-cognitive abilities have shown mixed results. Some researchers have found that students who use information technology more often tend to report higher levels of self-confidence, self-esteem, willpower, and internal control [44,53]. On the other hand, some studies suggest that IT use has little or even negative effects on these traits [54,55]. IT use can limit students’ chances to take part in real-life social interactions, such as talking, working together, or resolving problems face to face [56]. In addition, online environments often allow users to stay anonymous, and when students spend time on entertainment or social media under these conditions, they may become less sensitive to social rules. This lack of accountability may lead to a weaker sense of guilt, responsibility, or fear of consequences [57].

#### 2.2.3. Gaps in existing research and contributions of this study.

Recent studies from different perspectives have pointed out that using IT can help improve students’ cognitive and non-cognitive abilities. However, the findings are still under debate. Many existing studies rely on international large-scale survey data for sampling, and there is a lack of research focused on students in rural areas. These studies often apply experimental or quasi-experimental methods to assess the effects of providing hardware and software, while many others mainly use correlation analysis to examine patterns and levels of IT use. In addition, non-cognitive skills are not given enough attention in much of the existing literature.

Given this situation, this paper uses field survey data and a quasi-experimental design based on PSM-DID, using two waves of data collected from primary school students in rural western China. The goal is to better understand the current state of IT use among these students, estimate its effects on both cognitive and non-cognitive development, and explore differences across subgroups. A key feature of this study is that it goes beyond general measures of IT use by distinguishing three functionally different domains of digital engagement: learning, entertainment, and social interaction. This distinction is important because these forms of use differ in purpose, content, and likely developmental pathways, and may therefore generate heterogeneous effects on children’s cognitive and non-cognitive outcomes. This allows for a more detailed analysis of how different types of IT engagement may shape student outcomes, an area that has received little attention in previous research. The paper also attempts to analyze possible pathways through which IT may influence these outcomes. These findings aim to offer new evidence and practical guidance to support more effective, efficient, and beneficial strategies for developing human capital and promoting education informatization in underdeveloped regions.

## 3. Methods

### 3.1. Sampling and data collection

This study uses data from two waves of a longitudinal survey of third- and fourth-grade students in rural primary schools in western China. The data were collected by the Center for Experimental Economics in Education at Shaanxi Normal University. This study was conducted in accordance with the principles of the Declaration of Helsinki and was approved by the Institutional Review Board of the School of Economics and Management at Tsinghua University (protocol code 2019-TSEM-002, approval date: 15 November 2019). Data collection was carried out in December 2019 using written questionnaires, with approval obtained from the University. Written informed consent for participation and data use, including consent for publication of anonymized results, was obtained from schools, parents or guardians of participating students. All participants were fully informed that their anonymity would be preserved, the purpose of the research, the intended use of the data, and any potential risks associated with participation. No vulnerable individuals (e.g., patients, refugees) were included in the study. At the beginning of 2019, the research team conducted a preliminary survey in three cities in western China. In each city, seven counties were identified as research sites. Finally, 59 town-central primary schools across 21 counties were randomly selected as sample schools. In each selected school, one third-grade class and one fourth-grade class were randomly selected as the sample classes. The baseline survey was conducted in December 2019, and the follow-up survey was conducted in December 2020. Each survey included a student questionnaire, a standardized English test, and non-cognitive ability assessments. After excluding students who were lost to follow-up, the final sample consists of 3,235 students from 59 primary schools across 21 counties in western China, including only those students observed in both the baseline and follow-up surveys. Attrition between the two waves was minimal, suggesting that panel loss is unlikely to be a major source of bias in this study. Because this paper is based on a quasi-experimental design rather than randomized assignment, our main concern is the non-random selection into IT adoption. For this reason, the empirical analysis emphasizes balance between treatment and control groups after propensity score matching, rather than attrition-balance diagnostics commonly used in randomized trials.

In this study, the outcome variables include students’ cognitive and non-cognitive abilities. Cognitive ability is measured using their standardized English test scores, which reflect students’ academic development within the school setting. To evaluate non-cognitive ability, we apply a range of widely recognized psychological assessment tools. These include the Big Five Personality Test [58], the Grit Scale [59], the Internal-External Control Scale [60], the Mental Health Test (MHT), the Uncertainty Orientation Scale [61], and the Academic and Social Resources Self-Efficacy Scale [62].

Mental health is measured using the Mental Health Test (MHT), a widely used instrument for assessing children’s emotional and psychological difficulties in school settings. In our study, the MHT is treated as one dimension of students’ non-cognitive development because it captures emotional adjustment and self-regulation, which are closely related to children’s social-emotional functioning. The scale includes eight subdimensions: academic anxiety, people anxiety, loneliness tendency, self-blame tendency, hyper-sensitivity, health symptoms, terror tendency, and impulse tendency. The total MHT score ranges from 0 to 90, with higher scores indicating more severe psychological distress and poorer mental health. Accordingly, a positive coefficient on the MHT outcome should be interpreted as a deterioration in mental health rather than an improvement.

The key independent variable is students’ use of information technology, categorized into three domains: learning, entertainment, and socializing. This variable is defined by three survey questions:

(1) Have you used a learning machine, a computer, a mobile phone, or the Internet to study English this semester?
(2) Do you use a computer or mobile phone to access the Internet for entertainment (e.g., playing games, listening to music, watching videos)?
(3) Do you use a computer or mobile phone for social networking or chatting with friends?

Each question offers three possible responses: “often,” “sometimes,” or “never.” These response categories capture the frequency with which students participated in each type of IT activity, but they do not provide exact measures of duration (e.g., hours per day or week) or the amount of effort invested in the activity. Given the young age of the respondents and the need to maintain a simple and comparable questionnaire across rural schools, the survey was designed to measure domain-specific participation rather than precise usage intensity. In the main analysis, we therefore code “often” and “sometimes” as 1 (indicating participation in that domain of IT use) and “never” as 0 (indicating no participation). Accordingly, our treatment variables should be interpreted as indicators of whether students engaged in learning-, entertainment-, or social-oriented IT use, rather than as exact time-use or effort measures.

We control for a range of student characteristics at the individual, family, and school levels. At the individual level, these controls include gender (male = 1, female = 0), age, ethnicity (Han = 1, non-Han = 0), boarding situation (boarding = 1, not boarding = 0), and health situation (healthy = 1, unhealthy = 0). At the family level, these controls include whether the student has siblings (one or more siblings = 1; none = 0) and the parents’ education levels (mother’s and father’s education each coded as above junior high school = 1; junior high or below=0). Additionally, we include whether the mother works outside (yes = 1, no = 0), whether the father works outside (yes = 1, no = 0), and the family assets. At the school level, we include school fixed effects in the model to address potential endogeneity arising from differences across schools.

Table 1 provides a summary of the cognitive and non-cognitive development of all students in the sample, along with their individual and family background characteristics. On the whole, students show relatively low levels of cognitive development, with large differences between individuals. For non-cognitive traits, many students score well in areas like extraversion, agreeableness, conscientiousness, and openness. They also tend to show strong grit and are actively involved in school life. At the same time, some problems are noticeable, such as self-blame, hypersensitivity, and academic anxiety. The students in the sample vary widely in age. Around 40 percent are from ethnic minority backgrounds, and fewer than 10 percent are the only child in their families. Most of their parents have relatively lower levels of education. More than one-third of fathers work as migrant laborers, and the overall level of family assets is still low.

**Table 1:** Sample descriptives.

	N	Mean	SE	Min	Max
**Dependent variables: cognitive and non-cognitive abilities**					
**Cognitive ability (pre-test)**					
Standardized English test scores	3043	−0.064	1.026	−3.177	2.330
**Non-cognitive abilities (pre-test)**					
Total score of the Big Five Personality Test	3126	3.268	0.369	1	4.346
Extraversion	3124	3.209	0.529	1	5
Agreeableness	3123	3.497	0.633	1.500	5
Conscientiousness	3123	3.361	0.595	1.556	5
Neuroticism	3123	2.915	0.557	1	5
Openness to experience	3122	3.361	0.700	1.300	5
Grit degree	3125	3.215	0.502	1	5
Locus of control level	3235	8.418	3.774	0	18
Mental health	3235	43.87	17.20	0	90
Academic anxiety	3235	9.100	3.564	0	15
People anxiety	3235	5.109	2.561	0	10
Loneliness tendency	3235	3.909	2.469	0	10
Self-blame tendency	3235	5.863	2.673	0	10
Hyper-sensitivity	3235	5.583	2.529	0	10
Health symptoms	3235	6.260	3.413	0	15
Terror tendency	3235	4.685	2.834	0	10
Impulse tendency	3235	3.361	2.711	0	10
Academic self-efficacy	3118	3.620	1.081	1	5
Social resources self-efficacy	3097	7.509	2.528	0	10
School like	3235	6.113	2.395	0	9
School avoidance	3235	1.618	1.397	0	5
Like going to school	3058	8.427	2.480	0	10
Class like	3104	8.491	2.502	0	10
**Control variables**					
**Individual level**					
Gender (male = 1 and female = 0)	3215	0.508	0.500	0	1
Age	3192	9.946	1.219	8	17.25
Ethnicity (Han = 1 and non-Han = 0)	3235	0.591	0.492	0	1
Boarding situation (boarding = 1 and no boarding = 0)	3108	0.146	0.353	0	1
Health situation (health = 1 and unhealth = 0)	3119	0.702	0.457	0	1
Siblings (has one or more siblings = 1 and has no siblings = 0)	3087	0.914	0.280	0	1
**Family level**					
Mother’s education level (above junior high school = 1 and equal or below junior high school = 0)	3054	0.245	0.430	0	1
Father’s education level (above junior high school = 1 and equal or below junior high school = 0)	3052	0.276	0.447	0	1
Mother works outside (yes = 1 and no = 0)	3065	0.164	0.370	0	1
Father works outside (yes = 1 and no = 0)	3078	0.381	0.486	0	1
Family assets	3109	0.0350	1.414	−4.459	2.810

### 3.2. Treatment description

This study examines three domain-specific “treatments” that capture students’ adoption of information technology (IT) between the baseline survey (December 2019) and the follow-up (December 2020). At each wave, the student questionnaire asked whether the student (i) used a learning machine/computer/mobile phone/Internet for learning (e.g., online classes, homework, searching educational resources), (ii) used IT for entertainment (e.g., games, videos, music), and (iii) used IT for socializing (e.g., chatting/messaging, social networking). Response options were often/sometimes/never; we code often or sometimes as 1 (user) and never as 0 (non-user). Because our survey instrument identifies whether students adopted IT use in each domain but does not record exact time spent, the treatment captures domain-specific adoption between waves rather than the intensive margin of use.

For each domain, we construct a domain-specific panel indicator and restrict the sample to students who were non-users at baseline in that domain. For clarity, the treatment and control groups are defined separately for each IT-use domain. A student is classified as treated in a given domain if they were a non-user at baseline (0) and reported use at follow-up (1); students who remained non-users (0 → 0) serve as the control group for that domain. Because these domains reflect different purposes of IT, treatments are not mutually exclusive across domains (e.g., a student may adopt IT for learning but not for entertainment); therefore, we estimate three separate PSM-DID models (see 3.4), one per domain. Accordingly, the estimated effects should be interpreted as domain-specific adoption effects, rather than as mutually exclusive comparisons across all types of IT use. In other words, the coefficient for one domain captures the effect of adopting that specific type of IT use relative to remaining a non-user in that same domain, but does not imply that students in the treatment group do not also use IT for other purposes. Adoption is non-random; to proxy the selection process we estimate domain-specific propensity scores using rich baseline covariates: gender, age, ethnicity, boarding status, health status, only-child status/siblings, parental education, parental migration (mother/father works outside), family assets, baseline outcomes, and school fixed effects, and then match treated students to comparable controls prior to DID estimation.

### 3.3. Statistical models

#### 3.3.1. Ordinary least squares model.

The use of information technology (regarded as intervention) is hypothesized to improve students’ cognitive and non-cognitive abilities. This hypothesis is tested using an Ordinary Least Squares (OLS) regression model as follows:


$$\begin{array}{c} Y_{ijt}=\alpha +\beta_{\text{1}}\cdot {Tech}_{ijt}+\beta_{\text{2}}\cdot {Control}_{ijt}+\mu_{i} \end{array}$$ (1)

In equation (1), the dependent variable $Y_{ijt}$ represents cognitive and non-cognitive abilities of student i of school j at time t. The key independent variable, ${Tech}_{ijt}$, is a binary indicator denoting whether student i uses information technology for learning, entertainment, and socializing (1 if used, 0 if not used). The coefficient $\beta_{1}$ captures the impact of information technology use on students’ cognitive and non-cognitive abilities. The term ${Control}_{ijt}$ encompasses individual, family, and school-level control variables, while $\mu_{i}$ denotes the residual error term accounting for unobserved factors.

#### 3.3.2. PSM-DID model.

Students who did not use IT during baseline survey were divided into treatment and control groups. The treatment group received access to IT. During the follow-up survey, students in the treatment group had started using IT, while those in the control group still had no access.

However, the decision to use IT was not made randomly. It is likely that students’ personal traits and family background influenced whether they adopted IT or not. If we do not control for this selection bias, it becomes difficult to tell whether any observed differences in cognitive and non-cognitive outcomes are actually caused by IT use or by other unrelated factors. This limitation makes it hard to draw reliable conclusions about the effect of IT use on student performance.

To address this issue, this study adopts the Propensity Score Matching (PSM) method, drawing on the approaches proposed by Zheng, Ye and Chen [36,63,64]. First, we estimate each student’s likelihood of using IT through logistic regression, using a range of background variables. Because IT adoption was not random, students’ likelihood of using IT was influenced by baseline characteristics such as gender, age, ethnicity, health status, boarding status, parental education, parental migration status, and family assets. These covariates were included in the propensity score model to proxy the non-random selection mechanism. The regression model is specified as Eq. (2):


$$\begin{array}{c} \text{Pr}\left(D_{it\text{1}}\right)=\left(\alpha +\beta \cdot X_{it\text{0}}\right) \end{array}$$ (2)

where $D_{it1}$ is a dummy variable representing whether the respondent i received IT in the follow-up survey (1 = received; 0 = not received). $X_{it0}$ is a set of multidimensional indicators based on students’ individual and family levels. $t_{0}$ and $t_{1}$ represent periods before and after IT adoption.

Then, students from the treatment and control groups are matched based on their predicted probabilities. This matching process helps make the samples more similar, which greatly reduces the impact of selection bias.

Subsequently, a Difference-in-Differences (DID) approach is applied to compare changes in outcomes between matched treatment and control groups. The estimated impact is obtained by subtracting the differences observed in the control group from those observed in the treatment group. This method helps eliminate the influence of unobserved variables, thereby providing an accurate estimate of IT’s actual effect. The PSM-DID estimator is formalized as:


$$\begin{array}{c} {ATT}_{PSM-DID}=E\left[\overset{T}{\underset{t1}{Y}}-\overset{T}{\underset{t0}{Y}}|X_{t0},D=1\right]-E[\overset{C}{\underset{t1}{Y}}-\overset{C}{\underset{t0}{Y}}|X_{t0},D=0] \end{array}$$ (3)

In equation (3), T and C represent treatment and control groups (D = 1 for treatment, D = 0 for control), respectively. $t_{0}$ and $t_{1}$ represent periods before and after IT adoption. Variables $Y_{t0}$ and $Y_{t1}$ indicate the cognitive and non-cognitive ability scores of students before and after the change in information technology use (namely treatment), while $X_{t0}$ represents control variables. The results of the balance test between the treatment group and the control group before matching are presented in Supporting information (S1–S3 Table), and Supporting information (S4–S6 Table) display the differences between the two groups after matching. As can be observed, the groups exhibit no statistically significant differences after matching.

To address concerns regarding the plausibility of the parallel trends assumption with only two survey waves, we provide several supporting arguments. First, the balance tests (Supporting information, S1–S3 Table) indicate that, after propensity score matching, the treatment and control groups are statistically comparable in both baseline outcomes and observable characteristics. This suggests that the two groups started from a similar position before IT adoption. Second, the short time span between the baseline (2019) and follow-up (2020) surveys reduces the likelihood of unobserved shocks or major policy changes that could have systematically affected one group but not the other. Third, our model includes rich sets of individual, family, and school-level controls as well as school fixed effects, which further mitigate potential confounding. Finally, we conducted robustness checks using alternative matching strategies and outcome measures, and the results remain consistent. Taken together, these features enhance the credibility of the identifying assumption even though the formal pre-trend test cannot be implemented with only two periods.

#### 3.3.3. Heterogeneity model.

The impact of information technology (IT) use may differ across student groups with distinct characteristics. To examine this heterogeneity, individual and family characteristics, such as gender, age, ethnicity, boarding situation, health situation, siblings, parental education level, parental work outside, and family assets, are considered in this study. Specifically, the analysis investigates whether IT use has varying effects depending on students’ gender, age, maternal education level, and family assets.

The heterogeneity model employed is defined as follows:


$$\begin{array}{c} Y_{ijt}=\alpha +\beta_{\text{1}}\cdot {Tech}_{ijt}+\beta_{\text{2}}\cdot {Control}_{ijt}+\beta_{\text{3}}\cdot {(Tech}_{ijt}\cdot {Control}_{ijt})+\mu_{i} \end{array}$$ (4)

In equation (4), the interaction term ${Tech}_{ijt}\cdot {Control}_{ijt}$ represents the interaction between IT use (${Tech}_{ijt}$) and specific student characteristics (${Control}_{ijt}$). The coefficient $\beta_{3}$ captures the heterogeneous effects of IT use associated with these characteristics. The term $\mu_{i}$ represents the residual error capturing unobserved individual-level factors.

### 3.4. Research hypotheses

Based on the defined variables, we propose the following hypotheses, which will be tested through the subsequent model specification:

- Hypothesis 1: The use of IT in education can enhance students’ cognitive and non-cognitive abilities. Such use may broaden their horizons and stimulate their innovative thinking.
- Hypothesis 2: IT use in entertainment can hamper students’ cognition and non-cognition. IT use in entertainment distracts students to excessive visual/graphical content which impacts their developmental process.
- Hypothesis 3: Using IT for social interaction might also impair students’ cognitive and non-cognitive abilities. IT in socializing compromises students’ privacy and reduces opportunities for real-life interaction and cooperation.
- Hypothesis 4: The impact of IT use is more significant among Han nationality students. Due to differences in knowledge backgrounds and growth environments, Han students are better equipped to understand and utilize IT tools compared with non-Han students.
- Hypothesis 5: IT utilization will have a much bigger effect on older students, who are more capable of utilizing IT.
- Hypothesis 6: The effect of IT use may be greater for students whose mothers have lower levels of education. This is due to mothers with low education being less effective in affecting their children’s family education and, hence, making these students more vulnerable to the impact of IT use.
- Hypothesis 7: The impact of IT use is more significant for students from families with fewer assets. This is because limited family assets constrain educational opportunities, thereby amplifying the role of information technology in their learning.

## 4. Results

### 4.1. Effects of information technology use on cognitive and non-cognitive abilities

**Fig 1:**
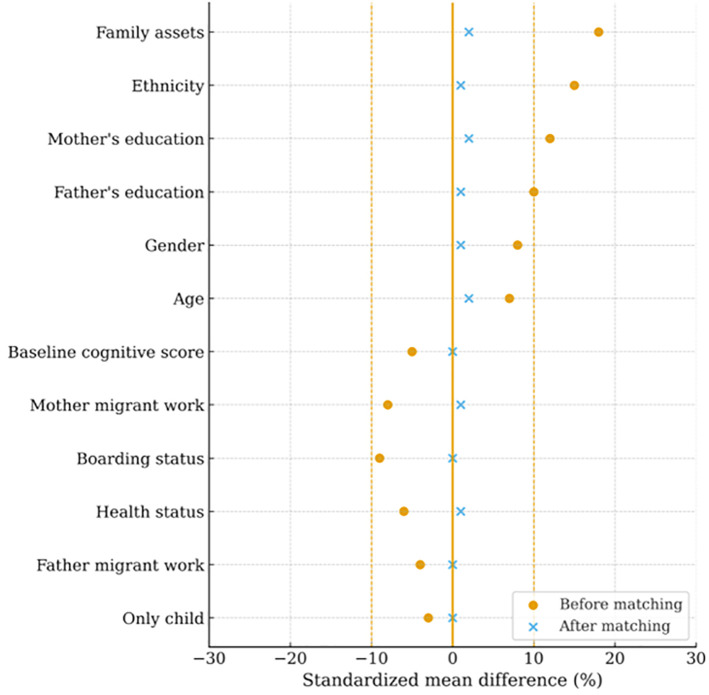
Standardized mean differences before and after matching for covariates: IT use in learning‌‌.

**Fig 2:**
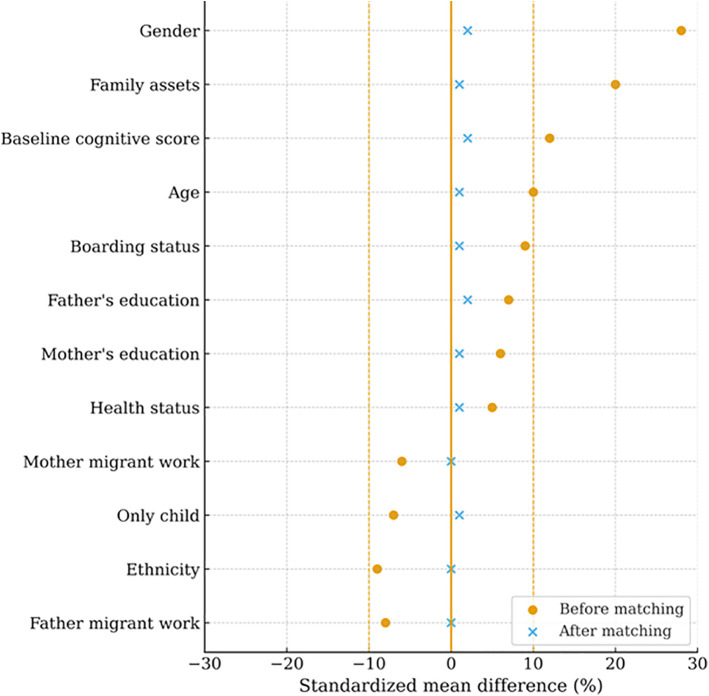
Standardized mean differences before and after matching for covariates: IT use in entertainment.

**Fig 3:**
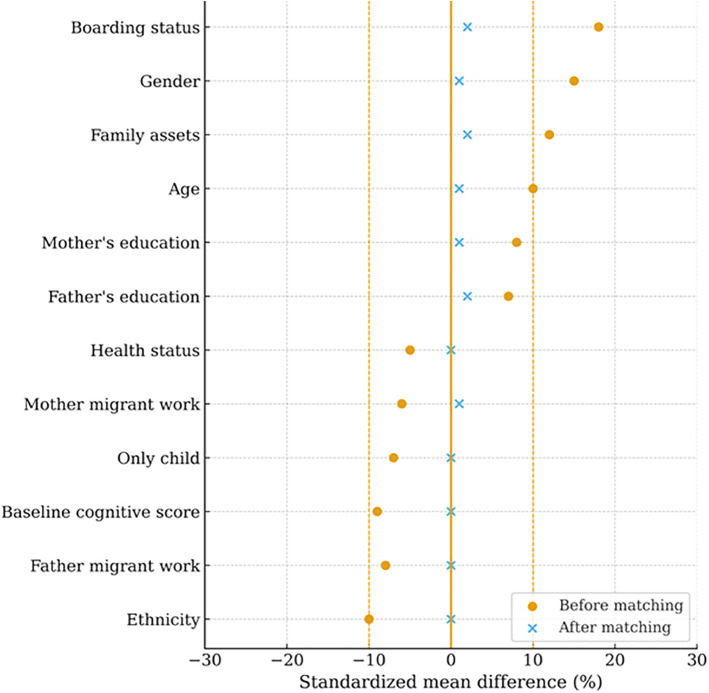
Standardized mean differences before and after matching for covariates: IT use in socializing.

S1–S3 Tables in supporting information present the covariate differences between treatment and control groups before matching, and S4–S6 Tables in supporting information show the corresponding results after matching. In all three IT-use domains, observable differences were effectively eliminated after matching. Figs 1–3 further illustrate that standardized mean differences were substantially reduced and remained well below the 10% threshold, indicating that covariate balance was achieved.

To ensure comparability, we used a kernel density matching method based on propensity scores to match students in the treatment and control groups. After matching, the results from the logistic regression showed no significant differences between the two groups across all observed characteristics, which indicates that the groups were sufficiently balanced. We then used the Propensity Score Matching Difference-in-Differences (PSM-DID) approach to estimate Equation (3). Table 2 presents the effects of IT use in three different domains, learning, entertainment, and socializing on students’ cognitive and non-cognitive outcomes.

**Table 2:** Effects of IT use on cognitive and non-cognitive abilities.

	(1)	(2)	(3)	(4)	(5)	(6)	(7)
	Standardized English test scores	Extraversion	Agreeableness	Conscientiousness	Neuroticism	Openness	Total score of the Big Five Personality Test
IT use in learning	−0.043	0.014	−0.036	−0.031	0.047	0.079**	0.017
	(0.032)	(0.034)	(0.040)	(0.033)	(0.032)	(0.036)	(0.020)
IT use in entertainment	0.016	0.006	−0.042	−0.108***	0.109***	0.040	−0.002
	(0.039)	(0.028)	(0.032)	(0.038)	(0.027)	(0.034)	(0.020)
IT use in socializing	−0.005	0.027	−0.081***	−0.047*	0.072***	0.083***	0.011
	(0.035)	(0.022)	(0.028)	(0.028)	(0.024)	(0.027)	(0.014)
All three dimensions	0.000	0.014	−0.041**	−0.037*	0.043***	0.041**	0.004
	(0.024)	(0.015)	(0.017)	(0.019)	(0.013)	(0.016)	(0.009)
	(8)	(9)	(10)	(11)	(12)	(13)	(14)
	**Grit degree**	**Locus of control level**	**Mental health**	**Academic anxiety**	**People anxiety**	**Loneliness**	**Self-blame tendency**
IT use in learning	0.005	0.377**	1.319*	−0.120	0.121	0.012	0.132
	(0.029)	(0.160)	(0.783)	(0.153)	(0.135)	(0.138)	(0.140)
IT use in entertainment	−0.116***	0.106	3.747***	0.499**	0.522***	0.535***	0.575***
	(0.035)	(0.175)	(0.924)	(0.193)	(0.130)	(0.168)	(0.155)
IT use in socializing	0.082***	0.231	1.904***	0.219*	0.265**	0.128	0.182*
	(0.024)	(0.139)	(0.618)	(0.125)	(0.106)	(0.122)	(0.094)
All three dimensions	−0.043**	0.190**	1.479***	0.189**	0.210**	0.136	0.194**
	(0.017)	(0.090)	(0.480)	(0.089)	(0.080)	(0.086)	(0.079)
	(15)	(16)	(17)	(18)	(19)	(20)	(21)
	**Hyper-sensitivity**	**Terror tendency**	**Impulse tendency**	**Academic self-efficacy**	**Social resources** **self-efficacy**	**Class like**	**Teacher like**
IT use in learning	0.458**	0.246*	0.372**	−0.002	0.058	0.257**	0.139
	(0.173)	(0.129)	(0.148)	(0.049)	(0.208)	(0.126)	(0.116)
IT use in entertainment	0.477***	0.523***	0.537***	−0.101**	0.603***	−0.494***	0.517***
	(0.164)	(0.162)	(0.146)	(0.040)	(0.147)	(0.136)	(0.135)
IT use in socializing	0.313**	0.202	0.584***	−0.048	−0.136	−0.394***	0.323***
	(0.124)	(0.124)	(0.117)	(0.039)	(0.131)	(0.105)	(0.100)
All three dimensions	0.264***	0.145*	0.312***	−0.050**	0.173*	−0.128*	0.182**
	(0.089)	(0.083)	(0.087)	(0.020)	(0.092)	(0.071)	(0.069)

Notes: 1. The characteristic variables in the model include gender, age, ethnicity, boarding situation, health situation, siblings, parental education level, parental work outside situation, family assets, baseline grades and school fixed effects. 2. The kernel matching strategy is used for propensity score matching. 3. * significant at 10%; ** significant at 5%; *** significant at 1%. Robust standard errors in parentheses.

The regression results show that using IT in any of the three domains does not lead to a clear improvement in students’ cognitive abilities. The estimated coefficients for cognitive outcomes are not statistically significant across learning (−0.043), entertainment (0.016), and socializing (−0.005). There may be two reasons for this. First, enhancing cognitive ability usually takes time and sustained effort. A one-year follow-up might not be long enough to observe meaningful changes. This is in line with Jaeggi et al. [65], who found similar results in a study conducted in the United States. Second, although IT can support learning, the potential benefits might be canceled out by its downsides. For example, frequent use of IT may reduce focus, lower study efficiency, and delay cognitive progress. This explanation is supported by previous studies, such as those by Cheema and Zhang and Falck et al. [66,67].

In contrast, IT use has more noticeable and varied effects on non-cognitive abilities. When used for learning, IT appears to improve students’ openness (0.079**), sense of control (0.377**), and liking for school (0.257**). However, using IT for entertainment shows negative effects. It lowers conscientiousness (−0.108***), reduces grit (−0.116***), and increases levels of anxiety (0.499**), impulsiveness (0.537***), and emotional sensitivity (0.477***). Similarly, IT use in social contexts lowers conscientiousness (−0.081***), raises anxiety (0.219*), and increases impulsivity (0.584***). These mixed outcomes suggest that IT may help develop open-mindedness and personal engagement, but at the same time, it can also bring about mental stress and behavioral problems. There are two likely explanations for these results. First, students at this age are still developing physically and mentally, and they may have difficulty understanding complex or harmful online content. This can negatively affect their personality development. Second, overuse of IT can reduce face-to-face communication with family and peers, which may lower their sense of responsibility and increase feelings of guilt. A plausible explanation is that learning-oriented IT differs from entertainment and social use in that it is embedded in goal-directed academic activities. Through access to new knowledge, interactive tasks, and feedback-rich learning environments, students may become more curious and more willing to engage with unfamiliar content, which is consistent with higher openness. At the same time, because such use is more closely connected to schoolwork and teacher-guided activities, it may reinforce students’ sense of connection to school and increase their positive feelings toward the learning environment.

These findings align with studies such as the randomized intervention by Banerjee et al. [50] in India and research by Ko et al. [57] in Taiwan, China. More broadly, this pattern is also consistent with recent research suggesting that the effects of children’s digital technology use are often more visible in engagement, well-being, and other non-cognitive dimensions than in short-term academic outcomes. Recent evidence further indicates that the consequences of digital use vary substantially by purpose and context, which may help explain why the estimated cognitive effects remain limited while the non-cognitive effects appear more mixed in our study. However, conflicting conclusions exist in the literature, including studies by Wittwer and Senkbeil [68] in Germany and Fiorini [55], which reported no significant relationship between IT use and non-cognitive abilities. Such inconsistencies likely result from differences in sample selection criteria, analytical methods, and definitions of IT usage. This study uniquely contributes by utilizing comprehensive survey data from rural western China, differentiating among various dimensions of IT use, and addressing specific aspects like duration and resource availability that have been overlooked in parts of the existing literature.

### 4.2. Robustness check

To ensure the accuracy and reliability of the main regression findings, this study conducts robustness tests by modifying the independent variable construction, altering the matching strategy used in propensity score matching, and employing alternative measures of cognitive abilities.

First, the robustness of findings concerning independent variables has been assessed by integrating IT usage across the three dimensions—learning, entertainment, and socializing—into a single composite measure using principal component analysis. Table 3 illustrates that the composite variable still yields an insignificant impact on cognitive ability, consistent with results in Table 2. Concerning non-cognitive outcomes, the composite measure shows significant effects, including decreases in conscientiousness (−0.037*) and increased neuroticism (0.043***), openness (0.041**), locus of control level (0.190**), mental health (1.479***), people anxiety (0.210**), hyper-sensitivity (0.264***), impulse tendency (0.312***), class like (−0.128*), and teacher like (0.182**). These results reinforce previous findings regarding the mixed non-cognitive effects of IT use.

**Table 3:** Robustness check 1&2: principal component analysis of IT use dimensions and alternative matching strategies.

	(1)	(2)	(3)	(4)	(5)	(6)	(7)
	Standardized English test scores	Extraversion	Agreeableness	Conscientiousness	Neuroticism	Openness	Total score of the Big Five Personality Test
Integrate three dimensions	0.000	0.014	−0.041**	−0.037*	0.043***	0.041**	0.004
	(0.024)	(0.015)	(0.017)	(0.019)	(0.013)	(0.016)	(0.009)
Nearest neighbor matching	−0.043	0.015	−0.038	−0.032	0.045	0.079**	0.018
	(0.032)	(0.034)	(0.040)	(0.033)	(0.032)	(0.036)	(0.020)
	(8)	(9)	(10)	(11)	(12)	(13)	(14)
	**Grit degree**	**Locus of control level**	**Mental health**	**Academic anxiety**	**People anxiety**	**Loneliness**	**Self-blame tendency**
Integrate three dimensions	−0.043**	0.190**	1.479***	0.189**	0.210**	0.136	0.194**
	(0.017)	(0.090)	(0.480)	(0.089)	(0.080)	(0.086)	(0.079)
Nearest neighbor matching	0.004	0.375**	1.331*	0.119	0.116	0.148	0.015
	(0.029)	(0.160)	(0.782)	(0.152)	(0.136)	(0.122)	(0.138)
	(15)	(16)	(17)	(18)	(19)	(20)	(21)
	**Hyper-sensitivity**	**Terror tendency**	**Impulse tendency**	**Academic self-efficacy**	**Social resources** **self-efficacy**	**Class like**	**Teacher like**
Integrate three dimensions	0.264***	0.145*	0.312***	−0.050**	0.173*	− 0.128*	0.182**
	(0.089)	(0.083)	(0.087)	(0.020)	(0.092)	(0.071)	(0.069)
Nearest neighbor matching	0.458**	0.241*	0.372**	−0.004	0.058	0.254**	0.139
	(0.173)	(0.128)	(0.148)	(0.049)	(0.208)	(0.126)	(0.115)

Notes: 1. The characteristic variables in the model include gender, age, ethnicity, boarding situation, health situation, siblings, parental education level, parental work outside situation, family assets, baseline grades, and school fixed effects. 2. The robustness test of principal component analysis uses kernel density matching, and the robustness test of the replacement matching strategy uses nearest neighbor matching. 3. * significant at 10%; ** significant at 5%; *** significant at 1%. Robust standard errors in parentheses.

Second, to check the robustness of the results, we replace the kernel density matching approach with the nearest neighbor matching approach (also in Table 3), which lead to the same result that do not show a strong improvement in any cognitive outcome in line with the previous conclusion. In terms of non-cognitive outcomes, the results show improvement in terms of openness (0.079**), locus of control level (0.375**), mental health (1.331*), hypersensitivity (0.458**), impulse tendency (0.372**), like for school (0.254**), and fear-related tendency (0.241*) retained strong relations, supporting the stability of the main results.

In the final check, shown in Table 4, we tested robustness by changing the way cognitive ability was measured. Instead of using standardized English test scores, we used students’ self-reported cognitive rankings in their class. Once again, IT use in learning (−0.031), entertainment (−0.052), and socializing (−0.021) does not show any significant effects on cognitive outcomes. This further supports the earlier conclusion that short-term IT use may not have a strong impact on cognitive development.

**Table 4:** Robustness check 3: alternative outcome using students’ self-reported cognitive ability.

	(1) IT use in learning	(2) IT use in entertainment	(3) IT use in socializing
Self-reported of cognitive ability	−0.031	−0.052	−0.021
	(0.053)	(0.070)	(0.070)
Pre-test score	0.343***	0.317***	0.340***
	(0.036)	(0.042)	(0.035)
Fixed effect	3.577***	3.550***	3.567***
	(0.047)	(0.052)	(0.050)
N	1,354	1,027	1,265

Notes: 1. The characteristic variables in the model include gender, age, ethnicity, boarding situation, health situation, siblings, parental education level, parental work outside situation, family assets, baseline grades and school fixed effects. 2. The kernel matching strategy is used for propensity score matching. 3. * significant at 10%; ** significant at 5%; *** significant at 1%. Robust standard errors in parentheses.

Taken together, the robustness checks confirm the above conclusions. While IT use does not appear to improve cognitive skills in the short term, it continues to show mixed and complicated effects on several non-cognitive dimensions.

### 4.3. Heterogeneity analysis

To explore whether the effects of information technology use in learning, entertainment, and socializing vary across different student subgroups, we estimate Equations (3) and (4) by including interaction terms. Table 5 reports the results across four key categories: ethnicity, mother’s education level, student age, and family asset level.

**Table 5:** Heterogeneity in IT use for different types of students.

	(1)	(2)	(3)	(4)	(5)	(6)	(7)	(8)
	Standardized English test scores	Total score of the Big Five Personality Test	Grit degree	Locus of control level	Mental health	Academic self-efficacy	Social resources self-efficacy	Class like
IT use in learning * Ethnicity	0.023	−0.055	0.007	0.181	3.182**	−0.024	−0.358	−0.049
	(0.064)	(0.040)	(0.060)	(0.378)	(1.545)	(0.093)	(0.356)	(0.266)
IT use in learning * Mother’s education level	−0.077	−0.080*	−0.050	−0.268	3.551	−0.209**	−0.302	−0.110
	(0.081)	(0.044)	(0.054)	(0.504)	(2.463)	(0.100)	(0.392)	(0.400)
IT use in learning * Age	0.009	0.051	0.026	0.132	−1.278	0.129	0.754**	−0.111
	(0.076)	(0.036)	(0.053)	(0.353)	(1.681)	(0.094)	(0.338)	(0.278)
IT use in learning * Family assets	−0.121	−0.014	−0.043	0.498	2.277	−0.002	−0.161	−0.254
	(0.078)	(0.045)	(0.056)	(0.451)	(1.804)	(0.101)	(0.385)	(0.254)
IT use in entertainment * Ethnicity	0.002	−0.062*	−0.151**	0.429	3.271**	−0.182*	−0.875***	−0.961***
	(0.083)	(0.037)	(0.060)	(0.351)	(1.622)	(0.099)	(0.233)	(0.283)
IT use in entertainment * Mother’s education level	0.073	0.036	−0.138*	0.440	4.765**	0.049	−0.254	0.030
	(0.069)	(0.046)	(0.076)	(0.315)	(2.185)	(0.094)	(0.336)	(0.321)
IT use in entertainment * Age	−0.026	−0.028	−0.178***	0.464	2.533*	−0.145*	−0.393	−0.358
	(0.070)	(0.032)	(0.060)	(0.360)	(1.445)	(0.080)	(0.260)	(0.300)
IT use in entertainment * Family assets	−0.053	0.014	−0.112**	−0.013	4.859***	−0.028	−0.651***	−0.558***
	(0.072)	(0.023)	(0.056)	(0.327)	(1.637)	(0.080)	(0.216)	(0.206)
IT use in socializing * Ethnicity	−0.002	−0.012	−0.071	0.265	1.130	−0.118	−0.129	−0.169
	(0.064)	(0.029)	(0.047)	(0.298)	(1.324)	(0.088)	(0.268)	(0.208)
IT use in socializing * Mother’s education level	−0.049	−0.039	−0.065	0.594*	1.784	−0.171**	−0.358*	−0.195
	(0.063)	(0.036)	(0.056)	(0.350)	(1.510)	(0.073)	(0.190)	(0.206)
IT use in socializing * Age	−0.059	0.012	−0.085	0.588*	1.897*	−0.056	0.052	−0.179
	(0.054)	(0.029)	(0.053)	(0.308)	(1.012)	(0.072)	(0.326)	(0.211)
IT use in socializing * Family assets	0.019	0.004	−0.058	0.311	2.892**	−0.022	−0.200	−0.389**
	(0.063)	(0.024)	(0.038)	(0.296)	(1.253)	(0.075)	(0.184)	(0.190)

Notes: 1. The characteristic variables in the model include gender, age, ethnicity, boarding situation, health situation, siblings, parental education level, parental work outside situation, family assets, baseline grades and school fixed effects. 2. * significant at 10%; ** significant at 5%; *** significant at 1%. Robust standard errors in parentheses.

#### 4.3.1. Ethnic differences.

From Table 5, we can see that the impact of IT use for learning on cognitive ability is not statistically significant for either Han or non-Han students. However, the estimated effect is slightly larger for Han students, suggesting that they might benefit more from learning supported by IT. One possible explanation is that many digital learning materials are developed with Han students’ cultural and language background in mind. These materials may feel more familiar and easier to understand for Han students, which could help them engage with the content more effectively.

As for non-cognitive outcomes, IT use in learning is linked to significantly higher mental health scores for Han students (3.182**), which reflects worse psychological well-being, since a higher score indicates more severe mental health problems. In contrast, non-Han students seem to be more affected by IT use in the context of entertainment. The data show that entertainment-related IT use significantly lowers their conscientiousness scores (−0.151**) and increases mental health concerns (3.271**). Moreover, it is associated with reduced academic self-efficacy (−0.875***) and lower levels of liking for school (−0.961***). These findings suggest that non-Han students may struggle to relate to digital content that does not align well with their cultural background. This mismatch might lead to weaker engagement and stronger negative emotional responses during IT use.

#### 4.3.2. Age differences.

In terms of cognitive ability, older students seem to benefit more from using IT for learning. Although the estimated coefficients are not statistically significant, the positive values suggest that older students may be better able to use IT tools for academic purposes. This could be related to their more developed thinking skills and greater familiarity with how digital resources can support schoolwork.

Regarding non-cognitive outcomes, the influence of IT use appears to vary across age groups depending on the context. For older students, using IT for learning is linked to a significant increase in academic self-efficacy (0.754**), which means they are more confident in their ability to perform well at school when supported by digital tools. On the other hand, younger students are more likely to be negatively affected by using IT for entertainment and social interaction. One notable finding is that entertainment-related IT use lowers their grit scores (−0.178***), and it is also related to mental health concerns. This may be because younger students are more drawn to entertaining digital content, which can easily capture their attention and influence their emotions, making them more vulnerable to distractions and potential negative effects.

#### 4.3.3. Maternal education level differences.

From the perspective of cognitive ability, students whose mothers have lower educational attainment show a slightly greater tendency to benefit from IT use in learning, although the results are not statistically significant. This pattern suggests that IT may serve as a supplementary educational tool in households where parents may lack the resources or knowledge to provide adequate academic support.

In the domain of non-cognitive abilities, IT use in learning decreases grit degree (−0.050) and academic self-efficacy (−0.209**) among students whose mothers have not completed nine years of compulsory education. This implies that IT access can partially compensate for the lack of parental educational support by fostering self-regulation and internal motivation. Conversely, IT use in entertainment negatively affects grit degree (−0.138*) in the same group, and contributes to worsening mental health as indicated by higher MHT scores, reflecting the potential for unguided digital engagement to undermine emotional well-being and self-discipline.

#### 4.3.4. Family asset level differences.

In terms of cognitive ability, students from low-asset families tend to be more negatively affected by IT use in learning, although these effects are not statistically significant. The general direction of the results suggests that financial limitations may prevent these households from making full use of digital learning tools in an effective way.

The patterns are more evident when looking at non-cognitive outcomes. For students from economically disadvantaged backgrounds, using IT for entertainment significantly increases their mental health scores (4.859***), which reflects poorer psychological well-being. It also lowers their grit levels (−0.112**) and leads to notable decreases in both academic self-efficacy (−0.651***) and social resource self-efficacy (−0.558***). In addition, using IT for social interaction is linked to a decline in how much these students like their classes (−0.389**). These findings suggest that students from low-income families are more vulnerable to the negative consequences of IT use. This underlines the importance of building supportive systems to help guide and regulate how technology is used in these settings, with the goal of promoting more equal access to educational benefits.

### 4.4. Discussion

Our findings on the educational impact of IT use are broadly consistent with trends reported in international research. When it comes to cognitive outcomes, we do not find any significant improvement in students’ test scores related to IT usage. This result aligns with many previous studies in both developed and developing countries, which also report either weak or no effects of computer access on student achievement [6,67,69]. For example, large-scale programs that provided students with home computers or internet access have often failed to show clear short-term gains in academic performance [68,70]. In some cases, the introduction of digital tools has even produced negative academic results. One well-known example is a Romanian voucher study, where students who received home computers saw a drop in grades, possibly due to distractions, even though their computer skills improved [69]. At the same time, our finding of no significant effect does not contradict earlier evidence that well-designed educational technology programs can be effective. Structured computer-based learning interventions have shown clear improvements in math and language scores in various settings, including urban India and Latin America [50,71]. The lack of a strong effect in our case may be due to how IT is used in the study setting. General exposure to technology, especially when used for multiple purposes, may not directly improve test performance. Even when students have access to valuable information or educational software, the benefits may be offset by time spent on non-academic digital activities, as discussed in earlier work [55].

Compared with the limited effects on cognitive outcomes, the influence of IT use on non-cognitive outcomes in this study is more obvious and shows a mix of both positive and negative results. Students who used IT more often became more open to new experiences and expressed a stronger liking for school, which suggests higher levels of engagement and emotional connection to their learning environment. At the same time, these students also reported higher levels of anxiety, self-blame, and other negative emotional reactions than their peers. This combination of positive and negative outcomes is similar to findings from international research on the relationship between technology use and students’ social and emotional development. Past studies have not reached a consistent conclusion about how IT affects non-cognitive abilities [66]. While some work has shown that using computers can help students build self-esteem and develop a stronger sense of control over their learning [72], other studies have raised concerns. For example, when students spend a lot of time on screens without clear guidance, they may interact less with others in real life, which can increase the risk of psychological problems [21,73]. The higher levels of anxiety observed in our study may reflect these risks and contribute to the wider concern about how unstructured technology use might affect children’s mental health. This pattern is also consistent with recent findings. Emerging evidence suggests that the developmental consequences of children’s digital technology use are highly heterogeneous across purpose and context, and that non-cognitive domains such as engagement, well-being, and self-regulation may respond more quickly than short-term academic performance. In this sense, the limited cognitive effects and the more mixed non-cognitive effects found in our study are not unusual, but instead reflect the broader complexity of children’s digital experiences.

Although this study focuses mainly on the estimated effects of IT use, it is also useful to consider the possible mechanisms behind these results. One reason why we do not find a significant effect on cognitive ability could be the “substitution effect”. In rural settings, where access to structured digital platforms is limited and parental supervision is often lacking, students may use IT more for entertainment than for learning. As a result, the time they spend on devices might take the place of activities like reading or doing homework [55].

Another explanation for the lack of clear cognitive benefits is the “masking effect”. This occurs when positive and negative impacts appear at the same time and cancel each other out. On the one hand, using IT for learning can provide useful content, interactive formats, and personalized learning paths supported by digital tools and data-based systems. These features may help with cognitive growth. On the other hand, without proper guidance, the advantages of IT can be outweighed by problems linked to entertainment or social media use. These include distractions and difficulty focusing. For younger students who are still building their basic thinking and emotional skills, the attraction of digital content may shift their attention away from schoolwork, leading to slower or more hidden negative effects over time.

Third, it is also possible that the short time frame of the study contributes to the results. Cognitive development usually happens gradually and requires long-term effort. Even though IT has the potential to support learning by giving better access to information or by encouraging critical thinking, such benefits might take time to show. The lack of change over one year may reflect a timing issue rather than an actual lack of impact. Studies that follow students for a longer period could offer better insight into these long-term effects.

In contrast, the mixed effects we see on non-cognitive outcomes may be linked to psychological or behavioral factors. A more specific explanation is needed for why learning-oriented IT, rather than entertainment- or social-oriented IT, is associated with improvements in some non-cognitive dimensions. One likely mechanism is that learning-oriented IT exposes students to a broader range of knowledge, problem-solving tasks, and unfamiliar ideas in a structured way. Such exposure may stimulate curiosity and exploratory thinking, which is closely related to openness to experience. A second mechanism is that learning-oriented IT is typically embedded in school-related activities, such as completing assignments, accessing educational materials, or interacting with teacher-directed content. Because of this close connection with formal learning, students may feel more engaged in classroom activities and more emotionally connected to school, which helps explain the positive effects on school liking and teacher liking. At the same time, the freedom that comes with using IT might boost students’ confidence, especially among those who feel comfortable with digital technology [74]. A third mechanism is that digital learning tools often provide immediate feedback, opportunities for self-paced learning, and a greater sense of autonomy in task completion. These features may strengthen students’ perceived competence and sense of control, which are important components of non-cognitive development. This interpretation is broadly consistent with the literature emphasizing the role of school belonging, self-efficacy, and supportive learning environments in shaping children’s social-emotional outcomes. However, when IT use becomes excessive or lacks structure, especially for entertainment, it can also create emotional pressure. Prior research shows that too much exposure to the internet is linked with higher anxiety levels and weaker emotional control [75].

Variability between subgroup effects in this scenario might capture differences in the degree of parental engagement, and thus digital literacy. To succeed in online learning environments with autonomy, students from a lower socioeconomic status face more difficulties by themselves, and consequently, the productive IT use on their behalf can be also less significant. These can thus tell us more about the role of an enabling context in moderating the influence of IT in fostering students’ learning.

## 5. Conclusion

This study uses baseline and follow-up survey data collected in 2019 and 2020 from third- and fourth-grade students in rural areas of western China to explore how IT use affects their cognitive and non-cognitive development. The main findings are as follows:

First, there are gaps in both cognitive and non-cognitive abilities among rural students in western China. On the one hand, many students demonstrate positive personality traits such as resilience and self-reliance. On the other hand, a large number of them also report high levels of learning anxiety, self-blame, and emotional sensitivity. These emotional problems are especially common among minority students and tend to be associated with factors like having siblings, wider age gaps within the family, low parental education levels, and limited household resources.

Second, while IT use does not have a significant effect on cognitive ability, the estimated direction is still positive. One possible explanation is that any cognitive benefits from IT access, including exposure to more information, interactive learning experiences, and tailored learning methods, may be offset by distractions from entertainment and social content. Although short-term gains in academic scores are not obvious, IT usage does appear to support non-cognitive development. It helps improve openness, strengthens students’ liking for school and teachers, and adds value to their learning experience. At the same time, this engagement may also increase emotional pressure, including anxiety, self-blame, and psychological discomfort. This shows that digital use among young students can have both benefits and risks.

Third, the analysis of different student groups reveals that IT has stronger effects on certain subpopulations. Older students, those of Han ethnicity, children whose mothers have lower levels of education, and students from lower family assets are more likely to be influenced by IT use. While digital access holds potential for reducing learning gaps for underprivileged groups, it is important to recognize the different needs across student backgrounds. Without careful design and support, technology could also widen existing inequalities. To make sure all students benefit from digital tools, tailored support and context-sensitive policies are necessary based on each group’s developmental and social conditions.

Based on the findings, we offer three policy suggestions. First, more attention should be given to using IT to promote educational fairness. IT can help students in less advantaged areas gain quicker access to learning opportunities, reducing the gap in access to quality education and narrowing regional differences in educational resources. To make this happen, investment in both technology and institutional support should be strengthened, especially in underdeveloped western regions and in areas with high minority populations. This includes improving digital infrastructure, upgrading teaching tools, and building better coordination systems that can support and improve the quality of IT-based learning. At the same time, digital learning interventions should be designed more flexibly across age groups. Since older students appear to benefit more from learning-oriented IT, schools may provide them with more structured and guided digital learning opportunities, while younger students may require closer supervision, simpler task arrangements, and stronger protection from distraction.

Second, while encouraging the benefits of IT use, it is also necessary to control its potential risks. Policymakers should continue to enhance protections for minors by blocking harmful online content and offering functions such as screen time limits and study mode settings suitable for young users. At the same time, families should take an active role in guiding children’s use of technology, by helping them develop healthy habits and setting examples for balanced and responsible IT use. This is particularly important for students from families with lower maternal education or fewer household assets, as these groups may be more vulnerable to the negative effects of unsupervised entertainment-oriented IT use. For such students, schools should strengthen home–school collaboration, provide parents with more specific guidance on effective and balanced IT use, and create supervised digital learning opportunities that reduce reliance on unguided digital entertainment.

Third, the different needs of student groups, especially those from minority backgrounds, need to be taken more seriously. Education authorities should design IT tools and services that match students’ cultural backgrounds and learning situations. Schools should also provide more specific and helpful guidance through classroom activities and family-school partnerships, so students can better deal with challenges related to their growth and emotional development. In particular, more effort should be made to develop culturally and linguistically appropriate digital resources for students from minority or culturally diverse backgrounds, so that the benefits of IT use are not concentrated only among students whose backgrounds are better aligned with mainstream digital content and learning platforms.

To sum up, these results suggest that simply increasing access to technology will not automatically improve students’ academic performance in the short term unless it is combined with proper guidance and thoughtful integration into teaching. However, IT use clearly has important effects on non-cognitive development, which can be both helpful and harmful. These effects vary across different types of students. Therefore, digital education efforts should be more flexible, targeted, and inclusive, especially when applied in rural areas or regions with fewer resources.

Although this study provides useful insights, it has a few limitations that should be acknowledged. First, both IT use and student outcomes were measured through self-reported questionnaires. It is widely recognized that self-reported data can be affected by bias. For instance, students may report their device usage or emotional experiences inaccurately, either by overestimating or underestimating them. These possible inaccuracies could influence the reliability of the results. In addition, our IT-use variables do not capture exact time spent or effort devoted to learning, entertainment, or social activities. The survey identifies whether students engaged in each domain and how frequently in broad categories, but not the intensive margin of use. As a result, our estimates should be interpreted as the effects of domain-specific IT participation/adoption rather than precise dose-response effects. Second, the research was conducted in primary schools located in rural areas of western China. The specific regional and population characteristics of the sample limit how much we can apply these findings to other contexts. Educational settings in cities or in other countries may show very different outcomes when it comes to the effects of IT use. For this reason, readers should be cautious in applying these conclusions beyond the current study area unless there is supporting evidence. Third, the study was carried out over a relatively short period, covering just one school year from the baseline to the follow-up survey. As a result, the effects we observed reflect only short-term changes after students started using IT. We still do not know whether the small cognitive changes or any of the shifts in non-cognitive development will continue, grow stronger, or fade away over time. Longer-term studies would be necessary to answer that question. A further limitation relates to the identification strategy. Because the study relies on two survey waves, we cannot formally test the parallel trends assumption that underpins the difference-in-differences approach. While this is a common limitation in quasi-experimental studies with short panels, we addressed it in several ways: by demonstrating baseline balance after propensity score matching, by controlling for extensive covariates and school fixed effects, and by confirming robustness across alternative specifications. Moreover, the study period was short and no major policy interventions were introduced in the sample schools, which makes it unlikely that systematic differences in outcome trajectories would have occurred independently of IT adoption. Nonetheless, we acknowledge this as a constraint and encourage future research with multi-wave longitudinal data to more directly examine pre-treatment trends.

Looking ahead, future research should investigate how various forms of IT engagement, especially in the context of AI-enhanced learning, impact both cognitive and socio-emotional development over time. Longitudinal studies and experimental designs are essential to capture delayed effects, while increased attention must be directed towards understanding how students’ developmental needs interact with the rapidly evolving digital environments they currently inhabit.

## Supporting information

S1 Table Treatment and control group before PSM: IT use in learning.
(DOCX)

S2 Table Treatment and control group before PSM: IT use in entertainment.
(DOCX)

S3 Table Treatment and control group before PSM: IT use in socializing.
(DOCX)

S4 Table Treatment and control group after PSM: IT use in learning.
(DOCX)

S5 Table Treatment and control group after PSM: IT use in entertainment.
(DOCX)

S6 Table Treatment and control group after PSM: IT use in socializing.
(DOCX)
