# Supplementary material for: Does digital access translate into human capital gains? Assessing information technology use effects on cognitive and non-cognitive development of students in Western Rural China
Source: PLoS One. 2026 Jun 1;21(6):e0349438. doi: 10.1371/journal.pone.0349438 (PMC13225661; doi:10.1371/journal.pone.0349438)
Supplement: S1 Table — Treatment and control group before PSM: IT use in learning. (DOCX) [file pone.0349438.s001.docx]

**Supporting information**

**S1 Table**

**Treatment and control group before PSM: IT use in learning**

|  | Treatment | | Control | | Difference in mean  (T-test) |
| --- | --- | --- | --- | --- | --- |
|  | N | Mean | N | Mean |  |
| **Dependent variables: cognitive and non-cognitive abilities** | | | | | |
| **Cognitive ability (pre-test)** | | | | | |
| Standardized English test scores | 826 | -0.071 | 656 | -0.130 | 0.059 |
| **Non-cognitive abilities (pre-test)** | | | | | |
| Total score of the Big Five Personality Test | 850 | 3.250 | 670 | 3.233 | 0.017 |
| Extraversion | 849 | 3.188 | 670 | 3.190 | -0.002 |
| Agreeableness | 849 | 3.497 | 670 | 3.448 | 0.049 |
| Conscientiousness | 850 | 3.351 | 669 | 3.337 | 0.014 |
| Neuroticism | 850 | 2.906 | 669 | 2.923 | -0.018 |
| Openness to experience | 850 | 3.305 | 668 | 3.260 | 0.045 |
| Grit degree | 849 | 3.223 | 670 | 3.202 | 0.022 |
| Locus of control level | 850 | 8.569 | 673 | 8.621 | -0.052 |
| Mental health | 850 | 44.481 | 673 | 45.964 | -1.483* |
| Study anxiety | 850 | 9.321 | 673 | 9.550 | -0.229 |
| People anxiety | 850 | 5.319 | 673 | 5.311 | 0.008 |
| Loneliness tendency | 850 | 4.046 | 673 | 4.137 | -0.091 |
| Self-blame tendency | 850 | 5.947 | 673 | 6.117 | -0.170 |
| Allergic tendency | 850 | 5.588 | 673 | 5.845 | -0.257** |
| Health symptoms | 850 | 6.105 | 673 | 6.547 | -0.442*** |
| Terror tendency | 850 | 4.864 | 673 | 4.920 | -0.056 |
| Impulse tendency | 850 | 3.292 | 673 | 3.538 | -0.246* |
| Academic self-efficacy | 847 | 3.568 | 671 | 3.586 | -0.018 |
| Social resources self-efficacy | 836 | 7.513 | 665 | 7.406 | 0.107 |
| School like | 850 | 6.211 | 673 | 6.180 | 0.031 |
| School avoidance | 850 | 1.636 | 673 | 1.768 | -0.132* |
| Like going to school | 825 | 8.412 | 650 | 8.472 | -0.060 |
| Class like | 842 | 8.501 | 659 | 8.458 | 0.043 |
| Teacher like | 838 | 8.291 | 660 | 8.303 | -0.012 |
| **Independent variable** | | | | | |
| Information technology use | 850 | 0 | 673 | 1 | -1.000 |
| **Control variables** | | | | | |
| **Individual level** | | | | | |
| Gender (male=1 and female=0) | 849 | 0.456 | 673 | 0.499 | -0.043* |
| Age | 843 | 9.886 | 668 | 9.887 | 0.000 |
| Ethnicity (Han nationality=1 and non-Han=0) | 850 | 0.535 | 673 | 0.621 | -0.086*** |
| Boarding situation (boarding=1 and no boarding=0) | 840 | 0.164 | 668 | 0.135 | 0.030 |
| Health situation (health=1 and unhealth=0) | 848 | 0.724 | 668 | 0.671 | 0.053** |
| Siblings (has one or more siblings=1 and has no siblings=0) | 839 | 0.927 | 658 | 0.897 | 0.031** |
| **Family level** | | | | | |
| Mother’s education level (above junior high school=1 and equal or below junior high school=0) | 829 | 0.192 | 646 | 0.251 | -0.059*** |
| Father’s education level (above junior high school=1 and equal or below junior high school=0) | 828 | 0.225 | 645 | 0.267 | -0.042* |
| Mother works outside (yes=1 and no=0) | 830 | 0.193 | 653 | 0.170 | 0.023 |
| Father works outside (yes=1 and no=0) | 837 | 0.389 | 654 | 0.349 | 0.041 |
| Family assets | 843 | -0.334 | 666 | -0.059 | -0.275*** |

Notes: * significant at 10%; ** significant at 5%; *** significant at 1%.
