# Supplementary material for: Does digital access translate into human capital gains? Assessing information technology use effects on cognitive and non-cognitive development of students in Western Rural China
Source: PLoS One. 2026 Jun 1;21(6):e0349438. doi: 10.1371/journal.pone.0349438 (PMC13225661; doi:10.1371/journal.pone.0349438)
Supplement: S2 Table — Treatment and control group before PSM: IT use in entertainment. (DOCX) [file pone.0349438.s002.docx]

**Supporting information**

**S2 Table**

**Treatment and control group before PSM: IT use in entertainment**

|  | Treatment | | Control | | Difference in mean  (T-test) |
| --- | --- | --- | --- | --- | --- |
|  | N | Mean | N | Mean |  |
| **Dependent variables: cognitive and non-cognitive abilities** | | | | | |
| **Cognitive ability (pre-test)** | | | | | |
| Standardized English test scores | 720 | -0.081 | 1369 | 0.037 | -0.118** |
| **Non-cognitive abilities (pre-test)** | | | | | |
| Total score of the Big Five Personality Test | 743 | 3.286 | 1,405 | 3.276 | 0.010 |
| Extraversion | 743 | 3.221 | 1,405 | 3.198 | 0.023 |
| Agreeableness | 743 | 3.559 | 1,405 | 3.514 | 0.045 |
| Conscientiousness | 742 | 3.385 | 1,405 | 3.371 | 0.014 |
| Neuroticism | 742 | 2.881 | 1,405 | 2.919 | -0.038 |
| Openness to experience | 742 | 3.381 | 1,404 | 3.380 | 0.001 |
| Academic self-efficacy | 742 | 3.239 | 1,405 | 3.232 | 0.007 |
| Social resources self-efficacy | 744 | 8.487 | 1,408 | 8.638 | -0.152 |
| Mental health | 744 | 43.816 | 1,408 | 45.553 | -1.737** |
| Study anxiety | 744 | 9.383 | 1,408 | 9.517 | -0.134 |
| People anxiety | 744 | 5.136 | 1,408 | 5.278 | -0.143 |
| Loneliness tendency | 744 | 3.819 | 1,408 | 4.032 | -0.213* |
| Self-blame tendency | 744 | 5.973 | 1,408 | 6.129 | -0.156 |
| Allergic tendency | 744 | 5.540 | 1,408 | 5.842 | -0.301*** |
| Health symptoms | 744 | 6.059 | 1,408 | 6.473 | -0.414*** |
| Terror tendency | 744 | 4.786 | 1,408 | 4.810 | -0.024 |
| Impulse tendency | 744 | 3.120 | 1,408 | 3.472 | -0.352*** |
| Grit degree | 741 | 3.707 | 1,404 | 3.632 | 0.074 |
| Locus of control level | 733 | 7.716 | 1,395 | 7.474 | 0.242** |
| School like | 744 | 6.437 | 1,408 | 6.320 | 0.117 |
| School avoidance | 744 | 1.550 | 1,408 | 1.656 | -0.107* |
| Like going to school | 719 | 8.606 | 1,374 | 8.452 | 0.154 |
| Class like | 735 | 8.774 | 1,395 | 8.556 | 0.218** |
| Teacher like | 730 | 8.564 | 1,393 | 8.445 | 0.119 |
| **Independent variable** | | | | | |
| Information technology use | 744 | 0 | 1408 | 1 | -1.000 |
| **Control variables** | | | | | |
| **Individual level** | | | | | |
| Gender (male=1 and female=0) | 744 | 0.379 | 1408 | 0.532 | -0.153*** |
| Age | 737 | 9.838 | 1399 | 9.957 | -0.119** |
| Ethnicity (Han nationality=1 and non-Han=0) | 744 | 0.663 | 1408 | 0.646 | 0.017 |
| Boarding situation (boarding=1 and no boarding=0) | 737 | 0.100 | 1400 | 0.117 | -0.017 |
| Health situation (health=1 and unhealth=0) | 737 | 0.708 | 1405 | 0.699 | 0.009 |
| Siblings (has one or more siblings=1 and has no siblings=0) | 728 | 0.922 | 1385 | 0.910 | 0.011 |
| **Family level** | | | | | |
| Mother’s education level (above junior high school=1 and equal or below junior high school=0) | 721 | 0.255 | 1360 | 0.248 | 0.007 |
| Father’s education level (above junior high school=1 and equal or below junior high school=0) | 718 | 0.266 | 1359 | 0.283 | -0.017 |
| Mother works outside (yes=1 and no=0) | 722 | 0.168 | 1372 | 0.152 | 0.015 |
| Father works outside (yes=1 and no=0) | 726 | 0.404 | 1379 | 0.350 | 0.053** |
| Family assets | 737 | -0.119 | 1399 | 0.091 | -0.210*** |

Notes: * significant at 10%; ** significant at 5%; *** significant at 1%.
