# Supplementary material for: Does digital access translate into human capital gains? Assessing information technology use effects on cognitive and non-cognitive development of students in Western Rural China
Source: PLoS One. 2026 Jun 1;21(6):e0349438. doi: 10.1371/journal.pone.0349438 (PMC13225661; doi:10.1371/journal.pone.0349438)
Supplement: S3 Table — Treatment and control group before PSM: IT use in socializing. (DOCX) [file pone.0349438.s003.docx]

**Supporting information**

**S3 Table**

**Treatment and control group before PSM: IT use in socializing**

|  | Treatment | | Control | | Difference in mean  (T-test) |
| --- | --- | --- | --- | --- | --- |
|  | N | Mean | N | Mean |  |
| **Dependent variables: cognitive and non-cognitive abilities** | | | | | |
| **Cognitive ability (pre-test)** | | | | | |
| Standardized English test scores | 1323 | -0.020 | 1305 | -0.087 | 0.066* |
| **Non-cognitive abilities (pre-test)** | | | | | |
| Total score of the Big Five Personality Test | 1,354 | 3.289 | 1,344 | 3.242 | 0.047*** |
| Extraversion | 1,354 | 3.225 | 1,343 | 3.179 | 0.045** |
| Agreeableness | 1,354 | 3.530 | 1,342 | 3.455 | 0.075*** |
| Conscientiousness | 1,353 | 3.392 | 1,343 | 3.328 | 0.064*** |
| Neuroticism | 1,353 | 2.911 | 1,344 | 2.920 | -0.009 |
| Openness to experience | 1,353 | 3.384 | 1,342 | 3.330 | 0.053** |
| Grit degree | 1,353 | 3.229 | 1,344 | 3.199 | 0.031 |
| Locus of control level | 1,354 | 8.519 | 1,349 | 8.867 | -0.348*** |
| Mental health | 1,354 | 44.160 | 1,349 | 46.720 | -2.560*** |
| Study anxiety | 1,354 | 9.312 | 1,349 | 9.585 | -0.273** |
| People anxiety | 1,354 | 5.218 | 1,349 | 5.417 | -0.199** |
| Loneliness tendency | 1,354 | 3.902 | 1,349 | 4.179 | -0.277*** |
| Self-blame tendency | 1,354 | 5.999 | 1,349 | 6.162 | -0.163* |
| Allergic tendency | 1,354 | 5.610 | 1,349 | 5.960 | -0.350*** |
| Health symptoms | 1,354 | 6.153 | 1,349 | 6.777 | -0.624*** |
| Terror tendency | 1,354 | 4.792 | 1,349 | 4.923 | -0.131 |
| Impulse tendency | 1,354 | 3.176 | 1,349 | 3.718 | -0.543*** |
| Academic self-efficacy | 1,350 | 3.657 | 1,342 | 3.586 | 0.071* |
| Social resources self-efficacy | 1,335 | 7.574 | 1,338 | 7.425 | 0.149 |
| School like | 1,354 | 6.312 | 1,349 | 6.274 | 0.039 |
| School avoidance | 1,354 | 1.569 | 1,349 | 1.782 | -0.213*** |
| Like going to school | 1,317 | 8.547 | 1,323 | 8.313 | 0.234** |
| Class like | 1,339 | 8.581 | 1,341 | 8.417 | 0.164* |
| Teacher like | 1,334 | 8.503 | 1,338 | 8.191 | 0.312*** |
| **Independent variable** | | | | | |
| Information technology use | 1354 | 0 | 1349 | 1 | -1.000 |
| **Control variables** | | | | | |
| **Individual level** | | | | | |
| Gender (male=1 and female=0) | 1354 | 0.460 | 1347 | 0.529 | -0.068*** |
| Age | 1344 | 9.895 | 1342 | 9.984 | -0.089* |
| Ethnicity (Han nationality=1 and non-Han=0) | 1354 | 0.653 | 1349 | 0.546 | 0.107*** |
| Boarding situation (boarding=1 and no boarding=0) | 1339 | 0.114 | 1342 | 0.179 | -0.065*** |
| Health situation (health=1 and unhealth=0) | 1346 | 0.713 | 1345 | 0.700 | 0.013 |
| Siblings (has one or more siblings=1 and has no siblings=0) | 1331 | 0.918 | 1331 | 0.913 | 0.005 |
| **Family level** | | | | | |
| Mother’s education level (above junior high school=1 and equal or below junior high school=0) | 1316 | 0.247 | 1314 | 0.235 | 0.012 |
| Father’s education level (above junior high school=1 and equal or below junior high school=0) | 1313 | 0.277 | 1315 | 0.264 | 0.013 |
| Mother works outside (yes=1 and no=0) | 1327 | 0.170 | 1316 | 0.163 | 0.006 |
| Father works outside (yes=1 and no=0) | 1327 | 0.396 | 1327 | 0.351 | 0.045** |
| Family assets | 1346 | -0.026 | 1336 | 0.067 | -0.093* |

Notes: * significant at 10%; ** significant at 5%; *** significant at 1%.
