# Supplementary material for: Does digital access translate into human capital gains? Assessing information technology use effects on cognitive and non-cognitive development of students in Western Rural China
Source: PLoS One. 2026 Jun 1;21(6):e0349438. doi: 10.1371/journal.pone.0349438 (PMC13225661; doi:10.1371/journal.pone.0349438)
Supplement: S4 Table — Treatment and control group after PSM: IT use in learning. (DOCX) [file pone.0349438.s004.docx]

**Supporting information**

**S4 Table**

**Treatment and control group after PSM: IT use in learning**

|  | Treatment | Control | P-value |
| --- | --- | --- | --- |
|  | Mean | Mean |  |
| N | 763 | 598 | - |
| Gender (male=1 and female=0) | 0.488 | 0.483 | 0.846 |
| Age | 9.924 | 9.917 | 0.914 |
| Ethnicity (Han nationality=1 and non-Han=0) | 0.612 | 0.607 | 0.870 |
| Boarding situation (boarding=1 and no boarding=0) | 0.137 | 0.138 | 0.978 |
| Health situation (health=1 and unhealth=0) | 0.684 | 0.687 | 0.913 |
| Siblings (has one or more siblings=1 and has no siblings=0) | 0.903 | 0.910 | 0.690 |
| Mother’s education level (above junior high school=1 and equal or below junior high school=0) | 0.246 | 0.234 | 0.631 |
| Father’s education level (above junior high school=1 and equal or below junior high school=0) | 0.266 | 0.253 | 0.611 |
| Mother works outside (yes=1 and no=0) | 0.172 | 0.169 | 0.893 |
| Father works outside (yes=1 and no=0) | 0.348 | 0.344 | 0.900 |
| Family assets | -0.059 | -0.083 | 0.754 |
| Standardized English test scores | -0.094 | -0.105 | 0.851 |
