# Supplementary material for: Does digital access translate into human capital gains? Assessing information technology use effects on cognitive and non-cognitive development of students in Western Rural China
Source: PLoS One. 2026 Jun 1;21(6):e0349438. doi: 10.1371/journal.pone.0349438 (PMC13225661; doi:10.1371/journal.pone.0349438)
Supplement: S5 Table — Treatment and control group after PSM: IT use in entertainment. (DOCX) [file pone.0349438.s005.docx]

**Supporting information**

**S5 Table**

**Treatment and control group after PSM: IT use in entertainment**

|  | Treatment | Control | P-value |
| --- | --- | --- | --- |
|  | Mean | Mean |  |
| N | 1271 | 668 | - |
| Gender (male=1 and female=0) | 0.529 | 0.512 | 0.386 |
| Age | 9.960 | 9.979 | 0.714 |
| Ethnicity (Han nationality=1 and non-Han=0) | 0.645 | 0.661 | 0.394 |
| Boarding situation (boarding=1 and no boarding=0) | 0.120 | 0.114 | 0.640 |
| Health situation (health=1 and unhealth=0) | 0.703 | 0.704 | 0.988 |
| Siblings (has one or more siblings=1 and has no siblings=0) | 0.913 | 0.911 | 0.858 |
| Mother’s education level (above junior high school=1 and equal or below junior high school=0) | 0.249 | 0.248 | 0.975 |
| Father’s education level (above junior high school=1 and equal or below junior high school=0) | 0.281 | 0.280 | 0.967 |
| Mother works outside situation (yes=1 and no=0) | 0.157 | 0.159 | 0.903 |
| Father works outside situation (yes=1 and no=0) | 0.351 | 0.345 | 0.745 |
| Family assets | 0.110 | 0.079 | 0.575 |
| Standardized English test scores | 0.049 | 0.053 | 0.926 |
