# Supplementary material for: Does digital access translate into human capital gains? Assessing information technology use effects on cognitive and non-cognitive development of students in Western Rural China
Source: PLoS One. 2026 Jun 1;21(6):e0349438. doi: 10.1371/journal.pone.0349438 (PMC13225661; doi:10.1371/journal.pone.0349438)
Supplement: S6 Table — Treatment and control group after PSM: IT use in socializing. (DOCX) [file pone.0349438.s006.docx]

**Supporting information**

**S6 Table**

**Treatment and control group after PSM: IT use in socializing**

|  | Treatment | Control | P-value |
| --- | --- | --- | --- |
|  | mean | mean |  |
| N | 1216 | 1241 | - |
| Gender (male=1 and female=0) | 0.527 | 0.523 | 0.840 |
| Age | 9.978 | 9.993 | 0.756 |
| Ethnicity (Han nationality=1 and non-Han=0) | 0.543 | 0.551 | 0.686 |
| Boarding situation (boarding=1 and no boarding=0) | 0.182 | 0.170 | 0.442 |
| Health situation (health=1 and unhealth=0) | 0.706 | 0.704 | 0.952 |
| Siblings (has one or more siblings=1 and has no siblings=0) | 0.914 | 0.913 | 0.982 |
| Mother’s education level (above junior high school=1 and equal or below junior high school=0) | 0.239 | 0.237 | 0.906 |
| Father’s education level (above junior high school=1 and equal or below junior high school=0) | 0.268 | 0.267 | 0.930 |
| Mother works outside (yes=1 and no=0) | 0.161 | 0.165 | 0.794 |
| Father works outside (yes=1 and no=0) | 0.347 | 0.357 | 0.617 |
| Family assets | 0.097 | 0.094 | 0.958 |
| Standardized English test scores | -0.070 | -0.065 | 0.893 |
